# Ethyl Acetate Fraction of *Hemerocallis citrina* Baroni Decreases Tert-butyl Hydroperoxide-Induced Oxidative Stress Damage in BRL-3A Cells

**DOI:** 10.1155/2018/1526125

**Published:** 2018-11-08

**Authors:** Jing Wang, Dongmei Hu, Jing Hou, Shushu Li, Weiping Wang, Jun Li, Jie Bai

**Affiliations:** Key Laboratory of Bio-Resource and Eco-Environment of Ministry of Education, College of Life Sciences, Sichuan University, Chengdu, 610065 Sichuan, China

## Abstract

The main purposes of this study were to screen the antioxidant activities of various fractions of *Hemerocallis citrina* Baroni and test their hepatoprotective effects in vitro. Antioxidant assays (2,2-diphenyl-1-picrylhydrazyl (DPPH), 2,2′-azino-bis(3-ethylbenzothiazoline-6-sulfonic acid) (ABTS), and reducing power experiments) and tert-butyl hydroperoxide- (t-BHP-) induced BRL-3A oxidative damage experiments were performed in vitro. The *H. citrina* ethyl acetate fraction (HCEA) was determined to have strong antioxidant activity because of its high flavonoid and polyphenol content. Ultraperformance liquid chromatography- (UPLC-) photodiode array (PDA)/mass spectrometry (MS) analysis showed that the main components of the HCEA were flavonoids and caffeic acid derivatives. A total of 17 compounds were identified. HCEA also effectively protected the liver against t-BHP-induced oxidative stress injury and significantly reduced reactive oxygen (ROS) accumulation. Moreover, HCEA significantly reduced levels of alanine aminotransferase (ALT), aspartate transaminase (AST), and lactate dehydrogenase (LDH). Further studies have shown that HCEA inhibits t-BHP-induced apoptosis by increasing B-cell lymphoma-2 (BCL-2) activity and decreasing caspase-3 and caspase-9 activity. Moreover, HCEA enhanced the activity of antioxidant enzymes superoxide dismutase (SOD) and catalase (CAT), as well as the total antioxidant capacity (T-AOC), and increased the antioxidant level of glutathione (GSH) in BRL-3A cells. HCEA increased the antioxidant capacity of cells by increasing the gene expression of AMP-activated protein kinase (*AMPK*), extracellular signal-regulated kinase (*ERK*), P38, nuclear factor, erythroid 2 like 2 (*Nrf2*), *SOD*, glutamate-cysteine ligase catalytic subunit (*GCLC*), glutamate-cysteine ligase modifier subunit (*GCLM*), and heme oxygenase 1 (*HO-1*), which are associated with antioxidant pathways to protect against oxidative stress. In conclusion, HCEA protected BRL-3A cells against t-BHP-induced oxidative stress damage via antioxidant and antiapoptosis pathways. Therefore, *H. citrina* Baroni may serve as a potential hepatoprotective drug.

## 1. Introduction

The liver is the major detoxification organ in the body. Liver diseases may be caused by recreational drug use [1], high-fat diet [2], excessive alcohol consumption [3], and viral infections [4]. Liver diseases pose serious threats to human health. Several previous studies have demonstrated that oxidative stress is the main etiological factor in various liver diseases [5, 6] because it destroys the antioxidant defense system [7, 8] and induces apoptosis [9, 10]. Therefore, reducing the accumulation of reactive oxygen species (ROS) may be an effective strategy to reduce liver damage induced by oxidative stress. The compound tert-butyl hydroperoxide (t-BHP) has been routinely used in the establishment of in vitro oxidative stress damage models [11, 12].

Many of the drugs prescribed in Western medicine have side effects and induce resistance. Therefore, interest in the research and development of hepatoprotective substances from natural products has increased. These substances have intrinsic antioxidant properties, and they directly or indirectly trigger intracellular signaling pathways to treat oxidative damage-related diseases [13].

*Hemerocallis citrine* Baroni (daylily) is a perennial herb of the Liliaceae family [14], which is indigenous to Asia, and its flowers are used for ornamental purposes and as food and medicine. According to previous studies, *Hemerocallis fulva* has been used to treat various diseases including depression [15], inflammation [16], insomnia [17], hepatosis [18], and cancer [19]. However, no studies have shown the hepatoprotective activity of *H. citrine* Baroni or any potential mechanism. Finally, the antioxidant activities of various fractions of *H. citrine* Baroni remain unknown. Therefore, the purpose of this study was to evaluate the antioxidant activity of *H. citrine* Baroni extracts and elucidate the mechanism of their hepatoprotective effects against t-BHP-induced oxidative damage in BRL-3A cells.

## 2. Materials and Methods

### 2.1. Reagents

Vitamin C (Vc), 2,6-ditert-butyl-4-methylphenol (BHT), 2,2′-azino-bis(3-ethylbenzothiazoline-6-sulfonic acid) (ABTS), and 2,2-diphenyl-1-picrylhydrazyl (DPPH) were purchased from Sigma-Aldrich Chemical Co. (St. Louis, MO, USA). Cell Counting Kit 8 (CCK-8) was purchased from KeyGen Biotech (Jiangsu, China). Alanine aminotransferase (ALT), aspartate transaminase (AST), total antioxidant capacity (T-AOC), superoxide dismutase (SOD), lactate dehydrogenase (LDH), glutathione (GSH), and bicinchoninic acid (BCA) protein quantification and cellular ROS detection assay kits were purchased from Nanjing Jiancheng Bioengineering Institute (Nanjing, China). The measurement kit for catalase (CAT) enzyme activity was purchased from Comin Biotechnology (Suzhou, China). The Annexin V-fluorescein isothiocyanate (FITC) and propidium iodide (PI) double staining assay kit was purchased from Vazyme Biotech (Nanjing, China). 4′,6-Diamidino-2-phenylindole (DAPI) was purchased from Solarbio (Beijing, China). Radio immunoprecipitation assay (RIPA) cell lysis buffer was purchased from NCM Biotech (Suzhou, China). TRIzol total RNA extraction kit was purchased from Zibo Biotech Co. Ltd. (Jiangsu, China). Reverse transcription kit was purchased from Takara Corporation Japan.

### 2.2. Extract Preparation

Daylily (*H. citrina* Baroni) was provided by Mingrun Agricultural Development Co. Ltd., Deyang, Sichuan, China. A voucher specimen was identified by Dr. Jie Bai, School of Life Sciences, Sichuan University, Sichuan, China. The powdered dry daylily flower (10 g) was extracted three times with 250 mL 70% ethanol under ultrasonication for 40 min at 59 HZ at 55°C. The filtrates were pooled and dried in a rotary vacuum evaporator. The crude extract was dissolved in distilled water and partitioned with n-hexane, ethyl acetate, and n-butanol six times per solvent using a separatory funnel. The *H. citrina* Baroni n-hexane (HCNH), ethyl acetate (HCEA), n-butanol (HCNB), and water (HCW) fractions were obtained and concentrated using a rotary vacuum evaporator and dissolved in 50% ethanol and serum-free medium for antioxidant activity determination and cell experiments, respectively.

### 2.3. Ultraperformance Liquid Chromatography- (UPLC-) Photodiode Array (PDA)/Mass Spectrometry (MS) Conditions

HCEA separation was performed using a Waters ACQUITY™ ultraperformance liquid chromatography (UPLC) system (Waters Corporation, Milford, MA, USA) equipped with a quaternary solvent manager system, a column compartment, an autosampler, and a photodiode array (PDA) detector. A 1 *μ*L aliquot of the sample solution was injected into a CORTECS UPLC T3 column (2.1 × 100, 1.6 *μ*m) maintained at 45°C. The autosampler temperature was maintained at 25°C. The mobile phase consisted of acetonitrile (A) and 0.1% (*v*/*v*) formic acid solution (B). It was delivered using the following optimized gradient program: 15%–25% A (0–4 min), 25%–35% A (4–5 min), 35%–15% A (5–5.01 min), and 15% A (5.01–8 min). The flow rate was 0.4 mL min^−1^ with a detection wavelength of 308 nm.

The Waters ACQUITY™ UPLC system was equipped with electrospray ionization (ESI) in the negative ion mode. The MS was operated using a capillary voltage of 0.8 kV, sample cone voltage of 15 V, desolvation temperature of 600°C, source temperature of 120°C, and desolvation gas flow of 240 L h^−1^. The data were collected from 150 Da to 700 Da using a 0.2 s scan time and uploaded to Empower v. 3.0 (Waters Corporation, Milford, MA, USA).

### 2.4. In Vitro Antioxidant Activity of HCEA

#### 2.4.1. Determination of Total Phenolic Content (TPC)

The total phenolic content of the HCEA fraction was determined using the Folin-Ciocalteu method according to a previously described method [20]. Briefly, a 10 *μ*L aliquot of the sample (1 mg mL^−1^) or gallic acid (0.016–0.5 mg mL^−1^) was mixed with 100 *μ*L Folin phenol reagent. After 5 min, 90 *μ*L 10% sodium bicarbonate (Na_2_CO_3_) was added, followed by mixing and incubation at 25°C for 40 min, and the absorbance at 765 nm was measured using a microplate reader (SpectraMax M2; Molecular Devices, Sunnyvale, CA, USA). The total phenolic content was determined using the linear equation method (*y* = 2.9001*x* + 0.076, coefficient of determination (*R* ^2^) = 0.998). The results are expressed as gallic acid equivalents (GAE).

#### 2.4.2. Determination of Total Flavonoid Content (TFC)

The total flavonoid content of the daylily extract fraction was determined by the colorimetric method as previously described [21]. Briefly, a 20 *μ*L sample (1 mg mL^−1^) or quercetin (0.008–0.5 mg mL^−1^) was mixed with 30 *μ*L of 5% NaNO_2_. After 6 min, 50 *μ*L of 10% AlCl_3_ was added. After 5 min, the mixture was added to 100 *μ*L of 10% NaOH and incubated at 25°C for 15 min. Absorbance at 510 nm was measured with a microplate reader. Total phenolic content was determined by the linear equation method (*y* = 0.5504*x* + 0.0489; *R* ^2^ = 0.9977). The results were expressed as quercetin equivalents (QE).

#### 2.4.3. DPPH Radical Scavenging Assay

DPPH radical scavenging activities were determined using a previously described method [22]. Briefly, 100 *μ*L aliquots of different sample concentrations were mixed with 100 *μ*L DPPH (0.1 mM) and incubated at 25°C for 30 min. The absorbance was measured at 517 nm using a microplate reader. Free radical scavenging capacity was calculated as follows: DPPH radical scavenging rate (%) = [1 − (Ai − As)/Ac] × 100, where Ai is the absorbance of the experimental group or positive control, As is the background sample absorbance, Ac is the absorbance of the negative control, and Vc is the positive control.

#### 2.4.4. ABTS Radical Scavenging Assay

The ABTS radical scavenging activities were determined using a published method [23]. Briefly, 100 *μ*L samples of different concentrations was mixed with 100 *μ*L diluted ABTS solution and incubated at 25°C for 30 min. The absorbance was measured at 734 nm using a microplate reader. The formula used to calculate the ABTS free radical scavenging activity was essentially the same as that used to determine the DPPH free radical scavenging activity with Vc as the positive control.

#### 2.4.5. Reducing Power Assay

The reducing power was measured according to a previously reported method [23]. In brief, 25 *μ*L samples of different concentrations, 50 *μ*L phosphate-buffered saline (PBS; pH 6.6, 0.2 M), and 25 *μ*L 0.1% potassium ferricyanide were mixed and incubated at 45°C for 1 h. Then, 50 *μ*L 10% trichloroacetic acid and 60 *μ*L 0.1% (*w*/*v*) ferric chloride were added to each mixture. The absorbance at 700 nm was measured using a microplate reader. BHT and Vc are positive controls.

### 2.5. Cell Culture

The normal rat liver cell line (BRL-3A) was obtained from the American Type Culture Collection (ATCC, CRL-1442; Manassas, VA, USA). The cells were cultured in Dulbecco's modified Eagle's medium (DMEM; Thermo Fisher Scientific, Waltham, MA, USA) containing 10% fetal bovine serum (FBS; Thermo Fisher Scientific, Waltham, MA, USA), 100 IU mL^−1^ penicillin, and 100 IU mL^−1^ streptomycin incubated at 37°C under a 5% CO_2_ atmosphere.

### 2.6. HCEA Toxicity Test Assay

CCK-8 was used to assess HCEA toxicity. When the cells had reached the logarithmic phase, they were transferred to 96-well plates at a density of 1 × 10^5^ cells mL^−1^. At ~50–60% confluence, the cells were exposed to various sample concentrations for 24 h, 10 *μ*L CCK-8 solution was added to each well, and the cultures were incubated for 1 h at 37°C. The absorbance was measured using a microplate reader. Complete uninoculated medium served as a blank control, and each experiment was replicated in five wells and repeated three times.

### 2.7. Protective Effects of HCEA against T-BHP-Induced Damage

BRL-3A cells were seeded at a density of 1 × 10^4^ and 2.6 × 10^5^ cells well^−1^ in 6- and 96-well plates. When the cells reached ~50–60% confluence, they were either left untreated or exposed to various sample concentrations and incubated for 24 h. Subsequently, the cells were either left untreated or treated with 500 *μ*M t-BHP solution for 1 h. The viability of cells in the 96-well plates was determined by adding 10 *μ*L CCK-8 to each well, followed by incubation for 1 h at 37°C. The absorbance was measured at 450 nm using a microplate reader. The cells in the six-well plate were treated with various drugs, washed twice with PBS, collected, and centrifuged, and then the apoptosis level and other indices were determined.

### 2.8. Measurement of ROS

According to previous reports, 2′,7′-dichlorodihydrofluorescein diacetate (DCFH-DA) has been used to analyze intracellular ROS levels [24]. Briefly, after exposure to t-BHP in a six-well plate, the cells were washed twice with PBS and treated with 5 *μ*M DCFH-DA. After staining in the dark for 30 min, the cells were observed under a fluorescence microscope (Olympus IX71, Olympus Corp., Tokyo, Japan) and photographed. Cells from another six-well plate were collected, stained, and analyzed using a BD FACSCalibur™ flow cytometer (BD Biosciences, Franklin Lakes, NJ, USA).

### 2.9. Apoptosis Assay

#### 2.9.1. DAPI Staining

BRL-3A cells were seeded into a 12-well plate at a density of 1.7 × 10^5^ cells well^−1^. After the cells were exposed to the drug, the medium was aspirated, and the cells were washed once with PBS and then fixed with 500 *μ*L 75% (*v*/*v*) ethanol for 12 h. The fixative was aspirated, the cells were washed once with PBS, 500 *μ*L 10 *μ*g mL^−1^ DAPI was added, and then the cells were stained in the dark for 15 min. They were then observed under a fluorescence microscope.

#### 2.9.2. Annexin V-FITC Staining Analysis of Apoptosis Using Flow Cytometry

An Annexin V-FITC apoptosis detection kit was used to detect apoptotic cells using flow cytometry according to the manufacturer's instructions [25]. Cells in a six-well plate were pretreated with various drugs, the medium was aspirated, and the cells were washed once with PBS. The cells were then collected by centrifugation at 300 × *g* for 6 min, the supernatant was discarded, and 200 binding buffer was added to the centrifuge tube. Then, 5 *μ*L each of Annexin V-FITC and PI was mixed with the cells, which were incubated in the dark at 23–25°C for 8 min, followed by the addition of 200 *μ*L Annexin V-FITC binding buffer, and the apoptotic cells were identified using flow cytometry.

#### 2.9.3. Quantitative Real-Time PCR (qRT-PCR) Analysis of Genes Related to Apoptosis

After the cells in the six-well plate were treated with specific drugs, 500 *μ*L TRIzol reagent was added to each well and the total RNA was extracted according to the commercial test kit instructions. RNA integrity was visualized and assessed using an agarose gel. cDNA was reverse-transcribed from the RNA using a reverse transcription (RT) kit. The quantitative real-time RT-polymerase chain reaction (qRT-PCR) was performed using the real-time SYBR Green method with a Bio-Rad CFX-96 thermocycler (Bio-Rad Laboratories, Hercules, CA, USA). After the reaction was completed, the nonspecific amplification and the primer dimer were excluded based on the dissolution curve. Glyceraldehyde 3-phosphate dehydrogenase (GAPDH) was used as the internal reference to convert the CT from the amplification using the 2^−△△Ct^ data analysis method.

### 2.10. Measurement of Key Enzymes in Cell Supernatants

The ALT, AST, and LDH activity levels in the cell supernatants were determined using commercial kits according to the instructions [26]. Absorbances were measured using a microplate reader, and the activities of AST, ALT, and LDH were expressed as units per liter (U L^−1^).

### 2.11. Determination of Antioxidant Activities

#### 2.11.1. Measurement of CAT, GSH, SOD, and T-AOC Antioxidant Levels

The antioxidant levels of CAT, GSH, SOD, and T-AOC were measured according to commercial test kit instructions [27]. Absorbance was measured using a microplate reader.

#### 2.11.2. qRT-PCR Analysis of Genes Related to Antioxidant Activities

The procedure followed here was the same as that described in Quantitative Real-Time PCR (qRT-PCR) Analysis of Genes Related to Apoptosis.

### 2.12. Statistical Analysis

Data are expressed as means ± standard error of the mean (SEM) of three independent experiments. The data were analyzed using GraphPad Prism 5 (GraphPad Software, San Diego, CA, USA). Significant differences between groups were determined using a one-way analysis of variance (ANOVA), and *P* < 0.05 indicated statistical significance.

## 3. Results

### 3.1. UPLC-PDA/MS Analysis of HCEA

**Figure 1:**
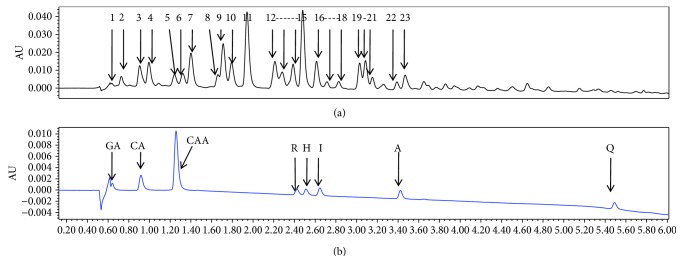
Ultraperformance liquid chromatography-photodiode array/mass spectrometry (UPLC-PDA/MS) analysis of *Hemerocallis citrina* Baroni ethyl acetate extract (HCEA). (a) Main chemical constituents in HCEA (1–23). (b) Peak map of standard product. GA: gallic acid; CA: chlorogenic acid; CAA: caffeic acid; R: rutin; H: hyperoside; I: isoquercitrin; A: astragalin; Q: quercetin.

Twenty-three major components were separated from HCEA using optimized UPLC (Figure 1(a)). The UPLC/PDA spectrogram revealed seven different caffeic acid derivatives (2–7, 11), fifteen different flavonoids (8–10, 12–23), and one phenolic compound (1). Seventeen of these compounds were identified by comparing the UPLC/MS output with reference standards and literature values (Table 1, Figure 1(b)). The main components of HCEA were found to be flavonoids and caffeic acid derivatives, which all have good antioxidant properties. Therefore, daylily could be used in the diet to enhance antioxidant and antiaging capacities.

### 3.2. In Vitro Antioxidant Assays

**Figure 2:**
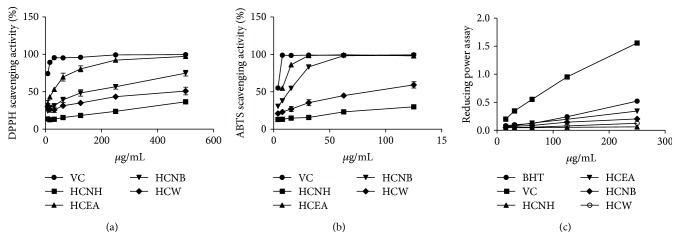
Antioxidant capacities of various extracts of *Hemerocallis citrina* Baroni: (a) DPPH scavenging radical assay; (b) ABTS scavenging radical assay; (c) reducing power assay. Data are expressed as means ± standard error of the mean (SEM, *n* = 3). DPPH: 2,2-diphenyl-1-picrylhydrazyl; ABTS: 2,2′-azino-bis(3-ethylbenzothiazoline-6-sulfonic acid); Vc: vitamin C; HCNH: *n*-hexane fraction; HCEA: ethyl acetate fraction; HCNB: *n*-butanol fraction; HCW: water fraction.

There was a positive correlation between antioxidant activity and total phenolic and flavonoid content [15, 16]. Our results indicated that HCEA had higher flavonoid and total phenolic levels than the other extract fractions did. The total flavonoid and phenolics in HCEA were 196.58 ± 0.015 mg g^−1^ QE equivalent and 102.86 ± 0.004 mg kg^−1^ GAE equivalent, respectively (Table 2). The three antioxidant assays showed that HCEA had higher antioxidant activity than the other extract fractions did (Figure 2). The half-maximal inhibitory concentrations (IC_50_, concentrations required to scavenge 50% of the radicals) were determined using GraphPad and are shown in Table 2. HCEA had strong antioxidant activity because of its high total flavonoid and phenolic content, and therefore, it was further analyzed in subsequent experiments.

### 3.3. Protective Effects of HCEA against T-BHP-Induced Damage

**Figure 3:**
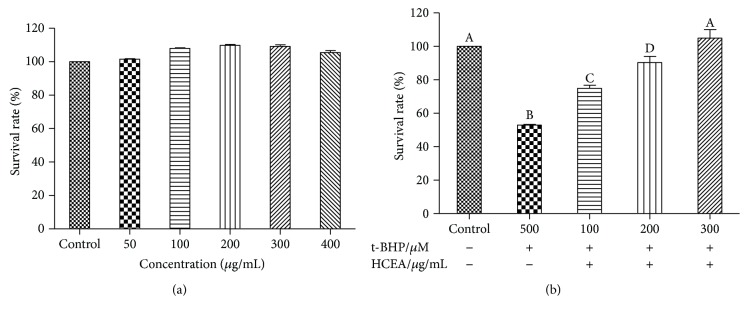
Cytotoxicity and cytoprotective effects of *Hemerocallis citrina* Baroni ethyl acetate fraction (HCEA). (a) BRL-3A cells exposed to various concentrations of HCEA (50–400 *μ*g mL^−1^) for 24 h. (b) BRL-3A cells pretreated for 24 h with indicated HCEA concentrations before treatment with 500 *μ*M t-BHP. Different lowercase letters indicate significant differences (*P* < 0.05).

When treated with 50–400 *μ*g mL^−1^ HCEA for 24 h, the BRL-3A cells had viabilities of 100%–110.63% (Figure 3(a)), which were not significantly different from that of the control group (*P* > 0.05). After treatment with only t-BHP, the cell viability was significantly reduced compared with that of the control group. After 24 h pretreatment with HCEA (100, 200, and 300 *μ*g mL^−1^), the BRL-3A cells were exposed to 500 *μ*M t-BHP for 1 h and their survival rates were significantly higher than those of the untreated cells were. Furthermore, these changes were concentration-dependent (Figure 3(b)) and, therefore, HCEA protected BRL-3A cells against t-BHP-induced oxidative stress.

### 3.4. HCEA Decreased Accumulation of ROS in BRL-3A Cells

**Figure 4:**
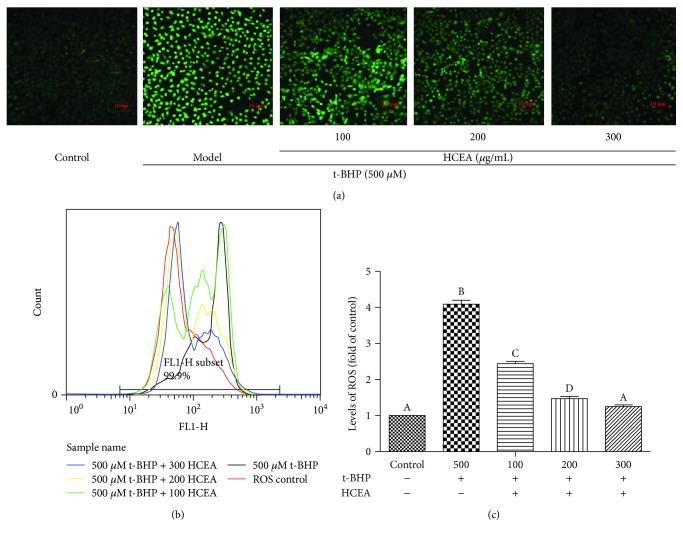
*Hemerocallis citrina* Baroni ethyl acetate fraction (HCEA) inhibits reactive oxygen species (ROS) production. Fluorescence of BRL-3A cells (a) observed under fluorescence microscope (20× magnification), (b) detected using flow cytometry, and (c) quantified. Different lowercase letters indicate significant differences (*P* < 0.05).

Intracellular ROS levels were analyzed using DCFH-DA, and pretreatment with HCEA significantly decreased t-BHP-induced ROS accumulation in a dose-dependent manner (Figure 4(a)). The flow cytometry showed that the intracellular ROS increased fourfold in the t-BHP-treated cells relative to the untreated control. However, intracellular ROS significantly decreased after HCEA pretreatment and the ROS levels of BRL-3A cells pretreated with 300 *μ*g mL^−1^ HCEA were reduced to the same as those of the control cells that were not exposed to t-BHP (Figure 4(b)). In conclusion, HCEA prevented t-BHP-induced intracellular ROS accumulation.

### 3.5. HCEA Inhibited T-BHP-Induced Apoptosis

**Figure 5:**
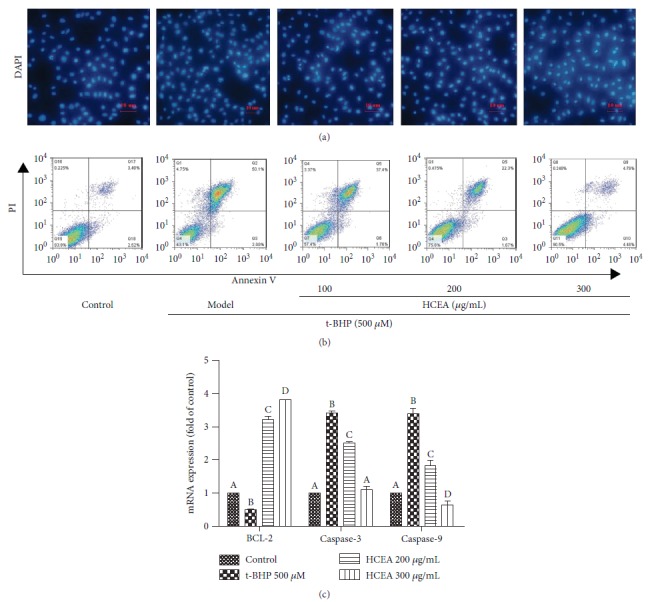
*Hemerocallis citrina* Baroni ethyl acetate fraction (HCEA) prevents t-BHP-induced apoptosis. (a) DAPI staining observed under a fluorescence microscope at 40× magnification. (b) Quantification of BRL-3A apoptotic cells using a flow cytometer with Annexin V-FITC/PI staining. (c) qRT-PCR analysis of expression of apoptosis-related genes. Different lowercase letters indicate significant differences (*P* < 0.05). DAPI: 4′,6-diamidino-2-phenylindole; qRT-PCR: quantitative real-time reverse transcription-polymerase chain reaction; FITC: fluorescein isothiocyanate; PI: propidium iodide.

After DAPI staining, morphological changes in the nuclei of the t-BHP-treated cells were observed under the fluorescence microscope. After t-BHP exposure, the nuclear chromatin collapsed, and the nuclei ruptured into fragments. In the control cells, however, the nuclei were intact, and the chromatin was uniform. In contrast, after HCEA pretreatment, the nuclear morphology gradually returned to normal in a concentration-dependent manner (Figure 5(a)). Moreover, pretreatment with specific HCEA concentrations (100, 200, and 300 *μ*g mL^−1^) prevented t-BHP-induced apoptosis (Figure 5(b)). Therefore, the protective effect of HCEA against t-BHP was antiapoptotic. Expression levels of the key genes involved in apoptosis were examined. As shown in Figure 5(c), the expression level of B-cell lymphoma-2 (*BCL-2*) decreased whereas those of caspase-3 and caspase-9 increased after t-BHP treatment. Therefore, t-BHP induced apoptosis in BRL-3A cells while HCEA pretreatment repressed the expressions of the apoptosis-related genes (caspase-3 and caspase-9) by inducing the expressions of the antiapoptotic gene *BCL-2*. These data show that HCEA protected BRL-3A cells against t-BHP-induced oxidative stress via the antiapoptosis pathway.

### 3.6. Key Enzyme Activity in Cell Supernatants

**Figure 6:**
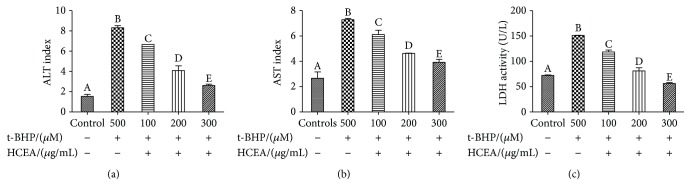
Key enzyme activities in cell supernatant. (a) ALT activity in cell supernatant, (b) AST activity in cell supernatant, and (c) LDH activity in cell supernatant. Different lowercase letters indicate significant differences (*P* < 0.05). ALT: alanine aminotransferase; AST: aspartate transaminase; LDH: lactate dehydrogenase.

Changes in ALT and AST activity reflect liver health status. In the present study, the activities of ALT and AST in BRL-3A cells treated with t-BHP were significantly higher than those in the corresponding control groups were. However, HCEA pretreatment significantly inhibited increases in ALT and AST in t-BHP-treated BRL-3A cells in a concentration-dependent manner (Figures 6(a) and 6(b)). Cells pretreated with HCEA also showed significantly reduced t-BHP-induced intracellular LDH levels relative to cells that were not pretreated (Figure 6(c)).

### 3.7. Protective Effects of HCEA on Antioxidant Activities

**Figure 7:**
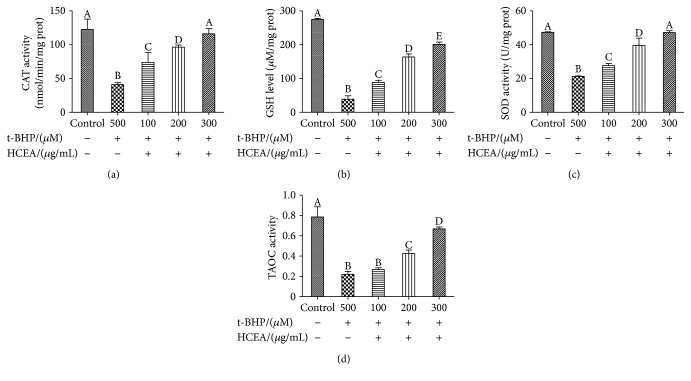
Effect of *Hemerocallis citrina* Baroni ethyl acetate fraction (HCEA) on antioxidant enzymes. (a) CAT activity in BRL-3A cell lysate; (b) GSH level in BRL-3A cell lysate; (c) SOD activity in BRL-3A cell lysate; (d) T-AOC activity in BRL-3A cell lysate. Different lowercase letters indicate significant differences (*P* < 0.05). CAT: catalase; GSH: glutathione; SOD: superoxide dismutase; T-AOC: total antioxidant capacity.

As shown in Figure 7, SOD, CAT, and TAOC activity significantly decreased in the t-BHP treatment group (*P* < 0.05) compared with those in the control group. HCEA pretreatment significantly increased the activity of these enzymes. HCEA also significantly increased the level of GSH to enhance antioxidant capacity. Therefore, HCEA protected the cells from oxidative damage by increasing their antioxidant enzyme activity and GSH levels. The protective effect of HCEA on BRL-3A cells subjected to t-BHP-induced oxidative stress was investigated by analyzing the antioxidant pathway and the expression of related genes such as *Nrf2*, *HO-1*, *GCLC*, *SOD*, *GCLM*, *AMPK*, *ERK*, and *p38*.

### 3.8. Effects of HCEA on Expression of Antioxidant Genes

**Figure 8:**
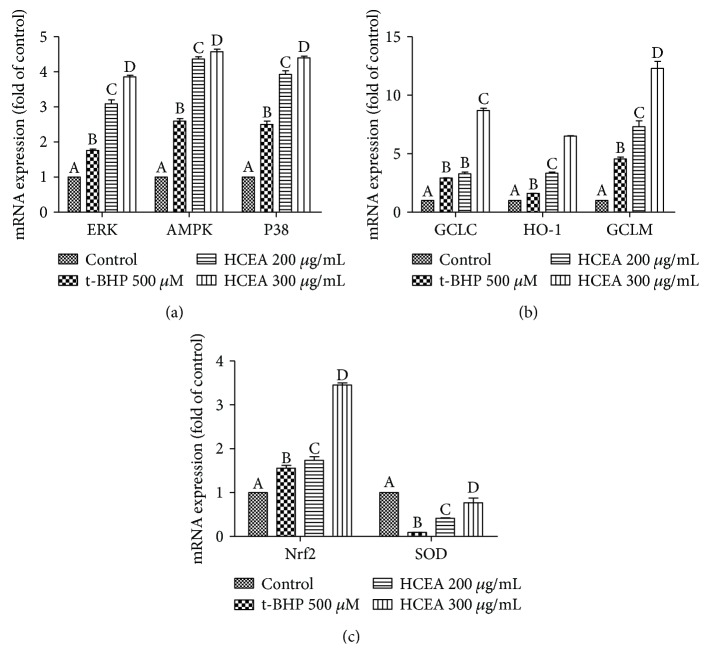
Expression of antioxidant-related genes. (a) Effect of HCEA on expression of ERK, AMPK, and P38. (b) Effect of HCEA on expression of GCLC, HO-1, and GCLM. (c) Effect of HCEA on expression of Nrf2 and SOD. Different lowercase letters indicate significant differences (*P* < 0.05). HCEA: *Hemerocallis citrina* Baroni ethyl acetate fraction; ERK: extracellular signal-regulated kinase; AMPK: AMP-activated protein kinase; GCLC: glutamate-cysteine ligase catalytic subunit; HO-1: heme oxygenase 1; CCLM: glutamate-cysteine ligase modifier subunit (GCLM); Nrf2: nuclear factor, erythroid 2 like 2; SOD: superoxide dismutase.

Oxidative stress induces the expression of antioxidant genes. When the antioxidant defense system activity is not sufficient to quench the overproduction of ROS, many diseases occur. In the current experiment, qRT-PCR analysis revealed that t-BHP treatment strongly inhibited the expression of SOD mRNA. After HCEA pretreatment, however, t-BHP-challenged cells expressed SOD mRNA normally (Figure 8(c)). As an oxidative stressor, t-BHP activates the antioxidant defense system in cells to protect cells from oxidative stress. As shown in Figure 8, when cells are treated with t-BHP, the antioxidant genes (*AMPK*, *P38*, *ERK*, *GCLC*, *GCLM*, *HO-1*, and *Nrf2*) were significantly increased, whereas cells pretreated with HCEA showed a significant increase in the expression of antioxidant genes compared with the model group cells. Therefore, HCEA prevented damage by intracellular oxidative stress. In conclusion, HCEA improved the antioxidant capacity of cells by enhancing the expression of antioxidant genes.

## 4. Discussion

Hydroxyl radicals, superoxide anion radicals, and various physiological and biochemical processes in the human body can produce ROS [10, 28, 29]. Very high ROS levels cause oxidative stress and, ultimately, cellular damage associated with apoptosis, cancer, aging, and the destruction of certain biomolecules [30, 31]. The use of natural substances with antioxidant properties may facilitate the prevention of diseases associated with oxidative damage [32].

In eastern Asia, the fresh and dried flowers of daylily have been widely used as a vegetable. We found, for the first time, that daylily has hepatoprotective activity and can be eaten as a health-enhancing vegetable, which is beneficial to human health. Furthermore, our findings provide a direction for further in-depth research. In the hepatoprotective test, the extracts were noncytotoxic at 400 *μ*g/mL and the survival rate was improved by 25% compared with the model group at 100 *μ*g/mL. This finding showed that HCEA has good hepatoprotective activity at 100 *μ*g/mL.

The results of the UPLC-PDA/MS analysis showed that the components of HCEA were mainly flavonoids and caffeic acid compounds, and the compounds 8, 13, 15, 16, 17, 19, 20, 21, 22, and 23 all contained glycosides. Studies have shown that the bioactivity of flavonoids after hydrolysis to aglycones is significantly higher than that of flavonoid glycosides. The potency of flavonoid aglycones is seven times that of flavonoid glycosides [33–36]. However, most glycosylated compounds are deglycosylated by the intestinal microbiota to glycosides, which is an essential step for effective intestinal absorption [37]. The concentration of the drug was slightly higher in the cell-based experiment because there were no hydrolyzing enzymes and intestinal microorganisms. In animals and humans, the presence of many enzymes and intestinal microbes changes the structure of the active ingredients and, thereby, the efficacy is increased.

Studies have shown that a bioactive polysaccharide TLH-3 isolated from *Tricholoma lobayense* protects against stress-induced premature senescence in cells and mice [38]. In the cellular experiment, the concentrations of TLH (100, 200, and 400 *μ*g/mL) showed significant protective effect, but the effect was already sufficient at the 200 mg/kg dose in the animal experiment [38]. In the experiment on the antioxidant and hepatoprotective effect of *Penthorum chinense Pursh* extract against t-BHP-induced liver damage in L02 cells [39], significant hepatoprotective effect was observed at a concentration of 200 *μ*g/mL, and excellent liver protection was observed in subsequent animal experiments. [40]. In the study of the ethyl acetate extract of the traditional Chinese medicine herb *Celastrus orbiculatus* against human gastric cancer, the *C. orbiculatus* ethyl acetate extract (COE) showed good inhibition of proliferation of AGS and BGC-823 cells at a concentration of 160 *μ*g/mL, while in animal experiments COE significantly inhibited tumor growth at a dose 40 mg/kg [41]. Other similar results [42, 43] showed a cellular concentration of >100 *μ*g/mL, which had a good effect in animal experiments. This may be related to some enzymes and digestion mechanisms in animals. Daylily is a plant of the genus *Hemerocallis*, which studies have shown to protect against alcohol-induced liver damage in mice [44]. The main component of *Hemerocallis* flavonoids is similar to that of daylily [44], indirectly indicating that daylily may potentially protect against alcohol-induced liver injury in mice. The present study showed that the daylily sample tested had relatively high levels of phenolics and flavonoids and strong antioxidant capacity. Therefore, the administration of daylily could reduce ROS production and oxidative stress damage, and it could be used as a potential hepatoprotective food.

Apoptosis is a normal process that removes damaged and tumorous cells [45]. However, cell apoptosis induced by oxidative stress could be detrimental [46]. Caspase-3 and caspase-9 are intracellular proenzymes that undergo proteolysis and participate in DNA repair and apoptosis [47]. Antiapoptotic proteins including BCL-2 are induced in response to oxidative stress, maintain mitochondrial membrane permeability, and prevent cytochrome release [48, 49]. In this study, we found that HCEA protected against t-BHP-induced cell injury by increasing BCL-2 activity and decreasing caspase-3 and caspase-9 activities, thereby enhancing antiapoptosis activity.

Certain liver enzymes such as ALT and AST are indicative of the liver health status. Changes in the expression levels of these enzymes may be associated with liver disease [50, 51]. Our results revealed that HCEA inhibited the activities of ALT, AST, and LDH. Therefore, daylily could be hepatoprotective.

Antioxidant enzymes such as SOD and CAT reduce oxidative stress-induced damage [10]. Previous studies reported that the transcription factor Nrf2 plays an important role in the antioxidant pathway [32]. Under normal conditions, Nrf2 and Kelch-like ECH-associated protein 1 (keep1) are bound in the cytoplasm; however, under oxidative stress, Nrf2 translocates to the nucleus and activates antioxidant genes such as *HO-1*, *GCLC*, *SOD*, and *GCLM* [8].

The nuclear transport factor Nrf2 often requires the activation of several signaling cascades such as mitogen-activated protein kinases (MAPKs) including ERK, c-Jun *N*-terminal kinase (JNK), and p38 [52]. t-BHP induces oxidative stress when it penetrates cells, and some protein kinase genes (*ERK*, *AMPK*, and *P38*) upstream of Nrf2 are activated, resulting in the transfer of Nrf2 from the cytoplasm to the nucleus, activating genes (*GCLC*, *GCLM*, and *HO-1*) involved in the synthesis of antioxidant enzymes to protect cells from oxidative stress. When ROS produced in the cells cannot be sufficiently removed by the antioxidant system, cells are damaged. Many studies [32, 53–56] have shown that cells treated with oxidative stress-inducing reagents only exhibit significant increases in antioxidant genes, but treatment with drugs further enhances the expression of these antioxidant genes, which enhances the protection from oxidative stress.

This phenomenon may be related to the defense state of the cell. When cells are subjected to oxidative stress, their antioxidant-related genes are elevated by their defense system to protect from oxidative stress. However, when the free radicals generated by oxidative stress are not sufficiently removed by the antioxidant system, the cells are damaged, resulting in diseases. The results of this study are also similar to the results reported in the literature. When treated with t-BHP, some of the genes involved in antioxidants are elevated, but pretreatment with HCEA can significantly increase the expression of these antioxidant genes, thereby more effectively protecting cells from oxidative stress.

## 5. Conclusions

HCEA had strong antioxidant activity, high total phenolic content, and high total flavonoid content. HCEA effectively protected BRL-3A cells against t-BHP-induced oxidative stress and reduced t-BHP-induced ROS accumulation by enhancing cellular antiapoptotic and antioxidant capacities. It enhanced BCL-2 expression, repressed caspase-3 and caspase-9, and increased cell survival. HCEA also improved the antioxidant capacity of cells by enhancing the expression of genes involved in the antioxidant pathway. UPLC-PDA/MS showed that the main components of HCEA were flavonoids and caffeic acid derivatives. A total of 17 compounds were identified. In summary, HCEA exhibited a strong cytoprotective effect and, therefore, *H. citrina* Baroni (daylily flowers) could potentially be used in the treatment of liver injury caused by oxidative stress.

## Figures and Tables

**Table 1:** Detection of compounds in *Hemerocallis citrina* Baroni ethyl acetate extract (HCEA) using ultraperformance liquid chromatography/mass spectrometry (UPLC/MS) in negative ion mode.

*N*	*t*_R_ (min)	Identification	[M-H]^−^	
			Indicated	Actual
1	0.658	Gallic acid	169.03	170.12
2	0.746	4-O-Caffeoylquinic acid	353.11	354.31
3	0.942	Chlorogenic acid	353.2	354.31
4	1.008	Not identified	325.03	_
5	1.258	Caffeic acid	179.03	180.15
6	1.362	4-O-p-Coumaroylquinic acid	337.18	338.309
7	1.442	1-Cyclohexene-1-carboxylic acid	335.17	336.3
8	1.708	Quercetin 3,7-O-*β*-D-diglucopyranoside	625.33	626.517
9	1.799	Not identified	479.12	_
10	1.849	Not identified	_	_
11	2.241	Not identified	449.12	_
12	2.295	Not identified	555.25	_
13	2.353	Hesperidin	609.31	610.561
14	2.441	Rutin	609.22	610.51
15	2.532	Hyperoside	463.12	464.3763
16	2.641	Isoquercitrin	463.1	464.38
17	2.761	Phloretin 2′-O-*β*-D-xylopyranosyl-(1-6)-*β*-D-glucopyranoside	579.27	580.12
18	2.9	Not identified	271.21	_
19	3.087	Quercetin 3-o-*β*-D-xylopyranoside	433.16	434.35
20	3.145	Kaempferol-3-O-galactoside	447.17	448.38
21	3.22	Kaempferol 3-rutinoside	593.22	594.52
22	3.438	Astragalin	447.09	448.37
23	3.508	Isorhamnetin 3-O-glucoside	477.15	478.4029

**Table 2:** Phenolic content, flavonoid content, and antioxidant activities of various extract fractions of *Hemerocallis citrina* Baroni.

Sample	Phenolic content (mg g^−1^)	Flavonoid content (mg g^−1^)	IC_50_ (*μ*g mL^−1^)		Reducing power
			DPPH	ABTS	
HCNH	14.55 ± 0.001	18.90 ± 0.084	5596 ± 3.748	1819 ± 0.182	78.94 ± 0.004
HCEA	102.86 ± 0.004	196.58 ± 0.015	20.82 ± 1.318	7.226 ± 0.009	4.497 ± 0.045
HCNB	63.31 ± 0.007	56.69 ± 0.004	115.4 ± 0.051	10.35 ± 0.032	8.025 ± 0.019
HCW	18.34 ± 0.095	53.23 ± 0.007	796 ± 0.215	80.58 ± 0.065	16.21 ± 0.015
Vc	nd	nd	3.12 ± 0.497	3.779 ± 0.003	0.9065 ± 0.148
BHT	nd	nd	nd	nd	2.954 ± 0.089

*H. citrina* Baroni extract fractions: HCNH: n-hexane; HCEA: ethyl acetate; HCNB: n-butanol; HCW: water; DPPH: 2,2-diphenyl-1-picrylhydrazyl; ABTS, 2,2′-azino-bis(3-ethylbenzothiazoline-6-sulfonic acid); nd: not detected.

## Data Availability

The primer sequences used in the qRT-PCR assay were all included in the supplemental material (available here). GAPDH as an internal reference gene. UPLC/PDA spectrogram of 23 major compounds of HCEA in the supplemental material. The UPLC/PDA spectrogram revealed seven different caffeic acid derivatives (2–7, 11), fifteen different flavonoids (8–10, 12–23), and one phenolic compound (1).

## Supplementary Materials

Supplementary materials S-Figure 1: UPLC/PDA spectrogram of 23 major compounds in HCEA. S-Table 1: primer sequences.
