# Supplementary material for: Ethyl Acetate Fraction of Hemerocallis citrina Baroni Decreases Tert-butyl Hydroperoxide-Induced Oxidative Stress Damage in BRL-3A Cells
Source: Oxid Med Cell Longev. 2018 Nov 8;2018:1526125. doi: 10.1155/2018/1526125 (PMC6250011; doi:10.1155/2018/1526125)
Supplement: Supplementary materials — S-Figure 1: UPLC/PDA spectrogram of 23 major compounds in HCEA. S-Table 1: primer sequences. [file 1526125.f1.doc]

S-table 1. Primer sequences

| Primer Nucleotide Sequences | | | Annealing  Temp (°C) |
| --- | --- | --- | --- |
| Caspase-3 | For. 5’: | GGATGGGTGCTATTGTGAGG | 60 |
|  | Rev. 5’: | TGGGATTTCAAGGCGACG |  |
| Caspase-9 | For. 5’: | CTTGTGTCCTACTCCACCTTC | 58 |
|  | Rev. 5’: | GTTAAAACAGCCAGGAATCTGC |  |
| Bcl-2 | For. 5’: | GTGGATGACTGAGTACCTGAAC | 60 |
|  | Rev. 5’: | CTTCACTTGTGGCCCAGATAG |  |
| ERK | For. 5’: | ACCAGCTCAACCACATTCTAG | 56 |
|  | Rev. 5’: | AAAGGTTAACATCCGGTCCAG |  |
| AMPK | For. 5’: | ATGGAATATGTGTCTGGAGGTG | 56 |
|  | Rev. 5’: | TTAGCATTCATCTGGGCGTC |  |
| P38 | For. 5’: | ATATTTGGTCCGTGGGCTG | 56 |
|  | Rev. 5’: | AGTTCATCTTCGGCATCTGG |  |
| GCLC | For. 5’: | TGGGAGTTACATGATTGAAGGG | 57 |
|  | Rev. 5’: | GTTGGGTCTGTGCTCTGG |  |
| GCLM | For. 5’: | CAAGAAAGCATCCCTGACATTG | 56 |
|  | Rev. 5’: | CCAGATAAATACAAGGCCCCTG |  |
| HO-1 | For. 5’: | CTTTCAGAAGGGTCAGGTGTC | 58 |
|  | Rev. 5’: | TGCTTGTTTCGCTCTATCTCC |  |
| Nrf-2 | For. 5’: | CAGAAGGAACAGGAGAAGGC | 58 |
|  | Rev. 5’: | GCATACAGTCTTCAAAGTACAAGG |  |
| SOD | For. 5’: | GGACAAACCTGAGCCCTAAG | 60 |
|  | Rev. 5’: | GCAATCTGTAAGCGACCTTG |  |
| GAPDH | For. 5’: | AACGACCCCTTCATTGACC | 60 |
|  | Rev. 5’: | CACGACATACTCAGCACCAG |  |


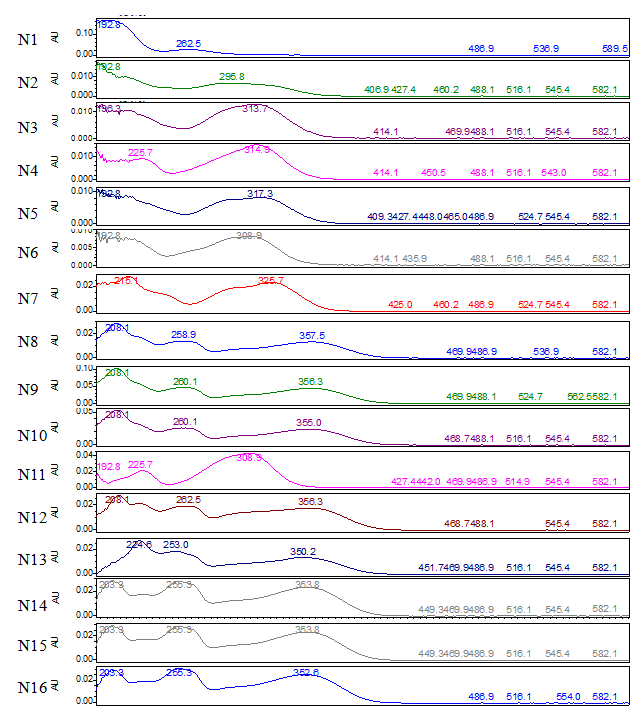


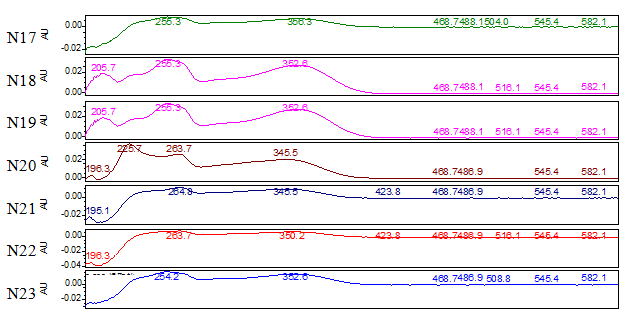


S-figure 1: UPLC/PDA spectrogram of 23 major compounds in HCEA.
